# Comparative transcriptome analysis reveals distinct ethylene–independent regulation of ripening in response to low temperature in kiwifruit

**DOI:** 10.1186/s12870-018-1264-y

**Published:** 2018-03-21

**Authors:** William O. Asiche, Oscar W. Mitalo, Yuka Kasahara, Yasuaki Tosa, Eric G. Mworia, Willis O. Owino, Koichiro Ushijima, Ryohei Nakano, Kentaro Yano, Yasutaka Kubo

**Affiliations:** 1 Graduate School of Environmental and Life Science, Okayama University, Okayama, 700–8530 Japan; 2 Meru University of Science and Technology, Meru, Kenya; 3 Department of Food Science and Technology, Jomo Kenyatta University of Agriculture and Technology, Nairobi, Kenya; 4 School of Agriculture, Meiji University, Kawasaki, 214–8571 Japan

**Keywords:** Ethylene, Fruit ripening, Low temperature–modulated ripening, On–vine ripening, Transcription factor

## Abstract

**Background:**

Kiwifruit are classified as climacteric since exogenous ethylene (or its analogue propylene) induces rapid ripening accompanied by ethylene production under positive feedback regulation. However, most of the ripening–associated changes (Phase 1 ripening) in kiwifruit during storage and on–vine occur largely in the absence of any detectable ethylene. This ripening behavior is often attributed to basal levels of system I ethylene, although it is suggested to be modulated by low temperature.

**Results:**

To elucidate the mechanisms regulating Phase 1 ripening in kiwifruit, a comparative transcriptome analysis using fruit continuously exposed to propylene (at 20 °C), and during storage at 5 °C and 20 °C was conducted. Propylene exposure induced kiwifruit softening, reduction of titratable acidity (TA), increase in soluble solids content (SSC) and ethylene production within 5 days. During storage, softening and reduction of TA occurred faster in fruit at 5 °C compared to 20 °C although no endogenous ethylene production was detected. Transcriptome analysis revealed 3761 ripening–related differentially expressed genes (DEGs), of which 2742 were up–regulated by propylene while 1058 were up–regulated by low temperature. Propylene exclusively up–regulated 2112 DEGs including those associated with ethylene biosynthesis and ripening such as *AcACS1*, *AcACO2*, *AcPL1*, *AcXET1*, *Acβ–GAL*, *AcAAT*, *AcERF6* and *AcNAC7*. Similarly, low temperature exclusively up–regulated 467 DEGS including *AcACO3*, *AcPL2*, *AcPMEi*, *AcADH*, *Acβ–AMY2*, *AcGA2ox2*, *AcNAC5* and *AcbZIP2* among others. A considerable number of DEGs such as *AcPG*, *AcEXP1*, *AcXET2*, *Acβ–AMY1*, *AcGA2ox1*, *AcNAC6*, *AcMADS1* and *AcbZIP1* were up–regulated by either propylene or low temperature. Frequent 1–MCP treatments failed to inhibit the accelerated ripening and up–regulation of associated DEGs by low temperature indicating that the changes were independent of ethylene. On–vine kiwifruit ripening proceeded in the absence of any detectable endogenous ethylene production, and coincided with increased expression of low temperature–responsive DEGs as well as the decrease in environmental temperature.

**Conclusions:**

These results indicate that kiwifruit possess both ethylene−dependent and low temperature–modulated ripening mechanisms that are distinct and independent of each other. The current work provides a foundation for elaborating the control of these two ripening mechanisms in kiwifruit.

**Electronic supplementary material:**

The online version of this article (10.1186/s12870-018-1264-y) contains supplementary material, which is available to authorized users.

## Background

Fleshy fruit ripening is a well–coordinated developmental process and involves physiological, biochemical and structural changes that are orchestrated by the expression of ripening–related genes through a network of signaling pathways [1]. Fruit are broadly classified into two groups (climacteric and non–climacteric) based on the presence or absence of a marked increase in respiration rate and ethylene production at the onset of ripening [2]. The ripening process is largely regulated by ethylene in climacteric fruit, while it is virtually independent of ethylene in non–climacteric fruit [2, 3]. Nevertheless, most fruit have been reported to possess both ethylene–dependent and –independent ripening components regardless of whether they are classified as climacteric or non–climacteric [4].

In climacteric fruit, ripening is accompanied by a shift from a negative feedback regulation (system I) to a positive feedback regulation (system II) of ethylene production [5], through ethylene–induced expression of *1–AMINOCYCLOPROPANE–1–CARBOXYLATE* (*ACC*) *SYNTHASE* (*ACS*) and *ACC OXIDASE* (*ACO*) [6, 7]. Ethylene then binds to ETHYLENE RESPONSE 1 (ETR1) and related proteins, transmitting the signal to ETHYLENE INSENSITIVE 2 (EIN2), ETHYLENE INSENSITIVE 3 (EIN3)/EIN3–LIKE (EIL) and later to ETHYLENE RESPONSE FACTORS (ERFs) in that order [8–10]. The ERFs modulate the transcription of a wide range of ripening–related genes that eventually result in fruit ripening responses [11]. Independent studies in tomatoes, kiwifruit, bananas and Arabidopsis have also revealed additional transcription factors (TFs) such as MADS–BOX, MYB, and NAM/ATAF1/2/CUC2 (NAC) that also regulate ethylene–dependent fruit ripening through interactions with one another [12–16]. In various climacteric fruit, application of 1–methylcyclopropene (1–MCP) suppressed ripening responses due to irreversible binding and phosphorylation of ethylene receptors [17–21]. Using 1–MCP, it was demonstrated that some ripening responses in tomato (such as peel de–greening and aroma biosynthesis) are regulated by ethylene, while other responses (such as sugar accumulation) are not always dependent on ethylene [22]. Moreover, Tassoni et al. [23] used the inhibition of ethylene response by 1–MCP to demonstrate that polyamines are not involved in tomato fruit ripening regulation. These results suggest that 1–MCP is a very useful tool in elucidating ethylene–independent fruit ripening responses.

Kiwifruit (*Actinidia* spp.) are considered climacteric since exogenous application of ethylene induces rapid ripening–associated changes [24]. However, most of the ripening–associated changes in kiwifruit occur before system II ethylene is produced [16, 25]. Furthermore, extensive ripening of kiwifruit in cold storage occurs in the absence of any detectable ethylene [20, 26, 27]. These unusual features of kiwifruit ripening are attributed to the basal levels of system I ethylene and/or changes in sensitivity, since kiwifruit are known to respond to ethylene levels as low as 0.01 μLL^− 1^ [28, 29]. However, there is no substantive research to ascertain this hypothesis due to lack of comparisons between ripening patterns of kiwifruit during cold and ambient temperature storage. The greatest challenge during storage of kiwifruit is the incidence of postharvest diseases (blossom–end rot, stem–end rot, and body rot) caused by pathogens such as *Botryosphaeria* sp., *Botrytis cinerea* and *Phomopsis* sp. [30, 31]. Infected fruit produce disease–induced ethylene, which may in turn induce ripening and feedback regulation of ethylene production in healthy adjoining kiwifruit [30]. Therefore, meaningful comparisons between the ripening behavior of kiwifruit during low and ambient temperature storage require the elimination of effects of exogenous ethylene emanating from disease–infected fruit.

Previously, we reported the faster softening and reduction of titratable acidity (TA) for ‘Sanuki Gold’ kiwifruit stored at 4 °C compared to 25 °C despite the lack of detectable ethylene production [32]. Accelerated fruit softening at 4 °C was accompanied by increased accumulation of *POLYGALACTURONASE* (*AcPG*), *PECTATE LYASE* (*AcPL*), and *EXPANSIN* (*AcEXP*) mRNAs. Repeated treatments of kiwifruit with 1–MCP failed to suppress the fruit ripening changes at 4 °C, and hence we speculated that they were modulated by low temperature independent of ethylene. However, exogenous propylene also induced the expression of *AcPG*, *AcPL* and *AcEXP*, providing a window of doubt on whether the expression of these genes during low temperature storage was independent of ethylene or not. Therefore, the question remains on whether basal ethylene is involved in the modulation of ripening in kiwifruit during cold storage or not.

Against this backdrop, the purpose of this study was to elucidate the mechanisms that regulate kiwifruit ripening during low temperature storage by comprehensive transcriptome analysis. In the first experiment, the aim was to monitor ethylene production patterns of kiwifruit to devise an appropriate technique for storage of kiwifruit at room temperature. This would enable us to provide meaningful comparisons with low temperature storage. Secondly, we conducted a comprehensive transcriptome analysis with comparisons between kiwifruit stored at either 5 °C or 20 °C for up to 8 weeks, relative to those exposed to propylene (an ethylene analogue). Thirdly, we monitored the ripening behavior of on–vine kiwifruit with detailed comparisons to stored fruit and those exposed to propylene. Based on these experiments, we concluded that low temperature modulates ripening of kiwifruit independent of ethylene. We discuss the role of low temperature as a newcomer in the modulation of fruit ripening both during storage and on the vine.

## Results

### Ethylene production pattern in postharvest kiwifruit

**Fig. 1:**
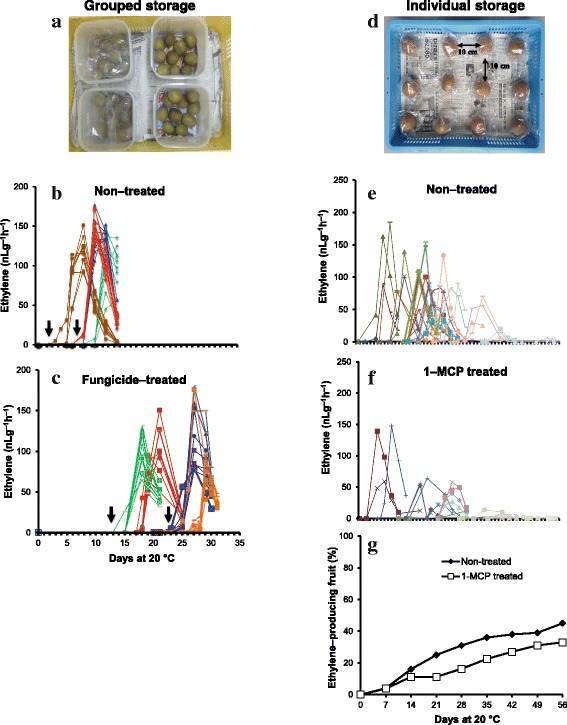
Ethylene production patterns of ‘Sanuki Gold’ kiwifruit as affected by storage technique. In grouped storage technique, kiwifruit at commercial maturity were divided into groups of ten, placed into containers and then covered lightly to reduce water loss (**a**). Four groups were stored at 20 °C without any treatment (**b**). Four other groups were pre–treated with a mixture of fungicides (0.015 g/L oxytetracycline, 0.15 g/L streptomycin, 0.5 g/L iprodione, 1 × 10^− 10^ cfu/L *Bacillus subtillis* HAI–0404 spores and 0.5 g/L benomyl) prior to storage and fortnightly during storage at 20 °C (**c**). Ethylene production pattern of individual fruit in each group was monitored periodically. Each line represents the ethylene production pattern of a single fruit. Lines of the same color represent fruit stored within the same container. In individual storage technique, kiwifruit at commercial maturity were pretreated with a mixture of fungicides and individually wrapped in perforated polythene bags before being placed in containers, about 10 cm apart (**d**). One group contained non–treated fruit (**e**), while another group of fruit were treated twice a week with 1–MCP at 5 μLL^− 1^ for 12 h (**f**). Fruit in all groups were stored at 20 °C in ethylene–free chambers. Ethylene production pattern of each fruit in the respective groups was monitored periodically. The proportion of ethylene–producing fruit was determined as a percentage of the total number of fruit in the respective groups (**g**)

At room temperature, postharvest kiwifruit show a synchronized burst of ethylene production within 6–25 d [10, 33–35], hindering possible comparisons of their ripening pattern with long–term cold storage. We speculated that the early and synchronized ethylene bursts observed in a batch of kiwifruit was due to ethylene emanating from adjoining diseased fruit. First, we monitored the ethylene production pattern of kiwifruit stored close to each other in the same container (grouped storage, Fig. 1a). Here, we observed that for all the four replications, kiwifruit stored in the same container exhibited a synchronized climacteric rise in ethylene production, although the occurrence of the climacteric ethylene peaks (7–13 d) differed for each group (Fig. 1b). Next, kiwifruit were treated with a mixture of fungicides to reduce the initial pathogen inoculum, and stored in groups (four replications). We observed a significant delay in ethylene production in fungicide–treated groups; synchronized climacteric ethylene peaks appeared between 19 and 30 d (Fig. 1c). In both non–treated and fungicide–treated groups, a few fruit initiated ethylene production 1–3 d prior to the others in the same group (see black arrows in Fig. 1b, c). These early ethylene–producing fruit exhibited rot symptoms (data not shown), suggesting that disease–infected fruit was the trigger of coordinated ethylene production in kiwifruit stored in groups.

Based on the above observations, we concluded that grouped storage is not appropriate for extending the postharvest life of kiwifruit at 20 °C. Instead, we treated kiwifruit with a mixture of fungicides and placed them in containers, ~ 10 cm apart from each other (individual storage, Fig. 1d). This setup consisted of non–treated and 1 − MCP treated fruit. Interestingly, we observed that the duration required for ethylene initiation and occurrence of climacteric peaks varied depending on each fruit (Fig.1e, f). While > 50% of fruit did not produce any detectable ethylene during the entire storage period (56 d at 20 °C), some fruit initiated ethylene production immediately after harvest, and others late during storage. The proportion of ethylene–producing fruit gradually increased during storage reaching ~ 45% and ~ 33% at 56 d for non–treated and 1–MCP treated fruit, respectively (Fig. 1g). Most of the ethylene–producing fruit developed rot symptoms within a few days after ethylene initiation, which was consistent with previous observations in grouped storage. Thus, the use of individual storage technique enabled us to single out and remove fruit producing ethylene due to disease infections; this is important to avoid accumulation of exogenous ethylene in storage chambers. As a result, we managed to obtain healthy kiwifruit showing no disease symptoms and no detectable ethylene for up to 56 days of storage at room temperature. These fruit were used for further comparisons with low temperature storage, as well as ethylene–dependent ripening.

### Effect of propylene on ripening in kiwifruit

**Fig. 2:**
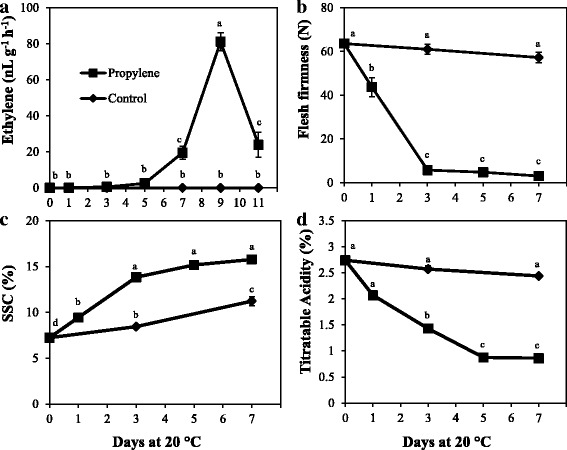
Ethylene–induced ripening in ‘Sanuki Gold’ kiwifruit. Kiwifruit at commercial maturity were divided into two groups. The first group was continuously exposed to propylene (5000 μLL^− 1^) at 20 °C to induce the ethylene effect. The second group was stored in air at 20 °C as a non–treated control. Ethylene production (**a**), flesh firmness (**b**), soluble solids content (**c**), and titratable acidity (**d**) were determined periodically using five independent biological replicates. Different letters indicate significant differences at *p* < 0.05

Continuous exposure of kiwifruit to propylene prompted an increase in ethylene production at 5 d, with a climacteric peak at 9 d (Fig. 2a). Flesh firmness of kiwifruit also decreased with propylene treatment, from 64 N at 0 d to < 10 N at 3 d (Fig. 2b). Furthermore, soluble solids content (SSC) increased rapidly from 7% at 0 d to > 15% at 5 d, and TA decreased from ~ 2.7% at 0 d to < 1% at 5 d in propylene–treated fruit (Fig. 2c, d). Non–treated fruit exhibited insignificant changes in ethylene production and flesh firmness while the changes in SSC and TA were much slower. Together, these results denote the ethylene–dependent ripening phenomenon in kiwifruit.

### Effect of storage temperature on ripening in kiwifruit

**Fig. 3:**
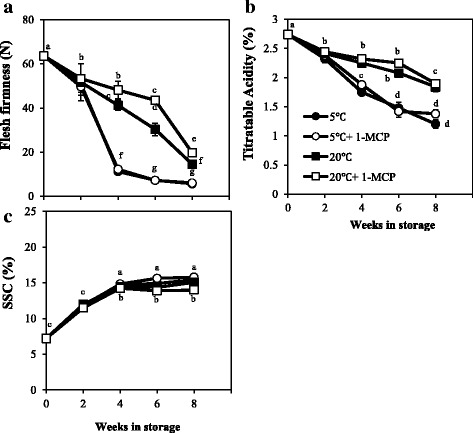
Changes in fruit ripening characteristics of ‘Sanuki Gold’ kiwifruit during storage at 20 °C and 5 °C. Kiwifruit at commercial maturity were divided into four groups. Each storage temperature had one group that was treated with 1–MCP, and another non–treated group. 1–MCP was applied twice a week at 5 μLL^− 1^ for 12 h. Fruit in all groups were stored in ethylene–free chambers using the individual storage technique (10 cm apart), and any fruit that produced detectable ethylene (> 0.01 nLg^− 1^ h^− 1^) were eliminated from storage chambers. Flesh firmness (**a**), titratable acidity (**b**) and soluble solids content (**c**) were determined using fruit that did not produce any detectable ethylene (five independent biological replicates). Different letters indicate significant differences at *p* < 0.05

To determine the effect of low temperature on ripening in kiwifruit, we stored kiwifruit at either 5 °C or 20 °C for up to 8 weeks in ethylene–free chambers. To avoid effects of exogenous ethylene, we used the individual storage technique and any fruit that produced detectable ethylene (> 0.01 nLg^− 1^ h^− 1^) was removed from the storage chambers. Therefore, only fruit that did not produce any detectable ethylene were used to monitor the changes in ripening characteristics. Fruit flesh firmness gradually decreased at similar rates during storage at both 5 °C and 20 °C for the first 2 weeks (Fig. 3a). Thereafter, fruit at 5 °C softened faster to an average flesh firmness of ~ 12 N and ~ 6 N at 4 and 8 weeks respectively, compared to ~ 40–48 N and 14–20 N for fruit at 20 °C at the same time–points. Similarly, fruit TA decreased at similar rates during storage at both temperatures for 2 weeks, and thereafter it decreased faster in fruit at 5 °C to ~ 1.8% and ~ 1.2% at 4 and 8 weeks respectively; kiwifruit at 20 °C maintained a high TA > 1.9% even after 8 weeks (Fig. 3b). At both 5 °C and 20 °C, kiwifruit SSC increased steadily from ~ 7% at harvest to > 14% at 8 weeks with insignificant differences between the two storage temperature conditions (Fig. 3c). The changes in firmness, TA and SSC were not suppressed by frequent 1–MCP treatments during storage at both temperature conditions. We also observed faster softening, TA decrease and SSC increase during storage of kiwifruit at 5 °C compared to 20 °C in ‘Rainbow Red’ and ‘Hayward’ cultivars (Additional file 1). These results indicate that in the absence of any detectable ethylene, low temperature accelerates softening and reduction of TA in kiwifruit.

### Overview of RNA sequencing analysis

**Fig. 4:**
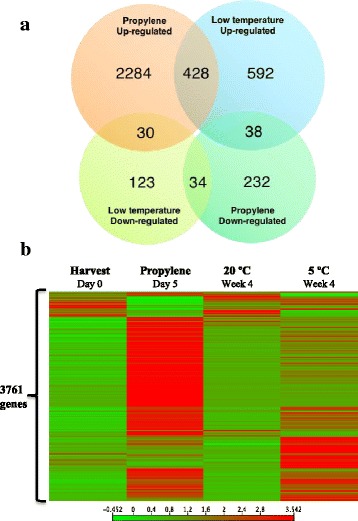
Comparison of transcriptome datasets between kiwifruit samples exposed to propylene and during storage. **a** Venn diagram for the differentially expressed genes (DEGs) in response to propylene and during storage at 5 °C. DEGs were selected based on a discriminatory criterion of > 3–fold change between propylene–treated/harvest, and 5 °C/20 °C samples. **b** A heat map showing the expression pattern of DEGs in kiwifruit at harvest (Day 0), after exposure to propylene for five days, and after storage at 20 °C and 5 °C with 1–MCP treatment for four weeks

To elucidate the ripening mechanisms in kiwifruit, we conducted a comprehensive transcriptome analysis using the Next Generation RNA sequencing technique. Comparisons were made between the transcriptomes of fruit at harvest (0 d) and those treated with propylene for 5 d, and between fruit stored at 5 °C and 20 °C for 4 weeks with 1–MCP treatment. The kiwifruit genome contains 39,041 annotated genes of which 3761 differentially expressed genes (DEGs) were identified (Fig. 4). Propylene treatment exclusively regulated 2516 DEGs, of which 2284 were up–regulated while 232 were down–regulated (Fig. 4a). Low temperature exclusively regulated 715 DEGs, of which 592 were up–regulated whereas 123 were down–regulated. We also identified 428 DEGs that were up–regulated, and 34 DEGs that were down–regulated by both propylene and low temperature. 30 DEGs were up–regulated by propylene while they were down–regulated by low temperature. By contrast, 38 DEGs were up–regulated by low temperature while they were down–regulated by propylene. Overall, propylene regulated most of the DEGs (3046), up–regulating 2742 DEGs and down–regulating 304 DEGs. Low temperature regulated 1245 DEGs, up–regulating 1058 and down–regulating 187. Detailed information about the DEGs with their annotation can be found in additional files 2–5. Next, we constructed a heat map to compare the RPKM values of the DEGs for fruit at harvest, after 5 d of propylene treatment and 4 weeks of storage at 5 °C and 20 °C (Fig. 4b). The expression patterns of the DEGs were quite similar for fruit at harvest and those after storage at 20 °C for 4 weeks. However, the patterns of propylene–treated fruit and those at 5 °C were completely different, indicating a significant change in their transcriptomes. Together, these results indicate that ripening in kiwifruit is orchestrated by three different sets of genes: the first set of genes is regulated by both ethylene and low temperature; the second set is exclusively regulated by ethylene, while the third set is exclusively regulated by low temperature.

### RT–qPCR validation of DEGs and expression analysis

**Fig. 5:**
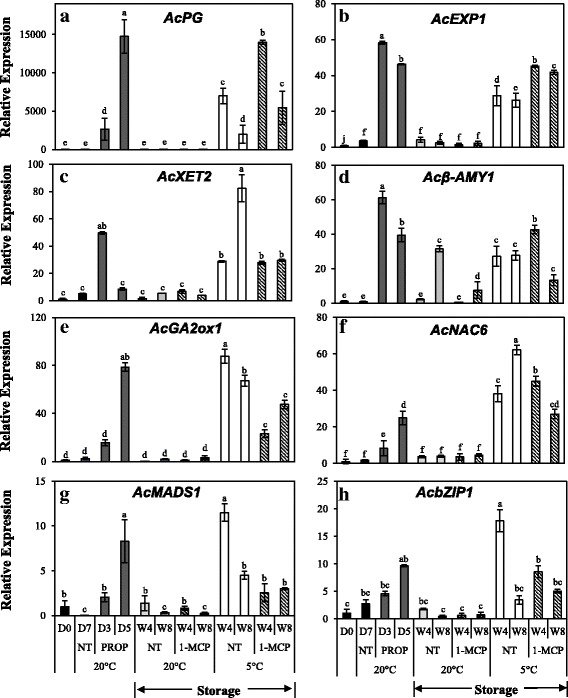
Reverse Transcriptase–Quantitative PCR analysis of selected kiwifruit genes that were up–regulated by both propylene and low temperature. Kiwifruit were continuously treated with 5000 μLL^− 1^ propylene (PROP) at 20 °C, alongside a non–treated group (NT). For storage, kiwifruit were kept at either 5 °C or 20 °C with (1–MCP) or without regular 1–MCP treatment (NT). 1–MCP was applied twice a week at 5 μLL^− 1^ for 12 h. Gene–specific primers were designed for (**a**) *AcPG*: *POLYGALACTURONASE* (Achn051381/AF152756); (**b**) *AcEXP1*: *EXPANSIN 1* (Achn336951/AY390358); (**c**) *AcXET2*: *XYLOGLUCAN ENDOTRANSGLUCOSYLASE 2* (Achn38797); (**d**) *Acβ–AMY1*: *β–AMYLASE 1* (Achn141771/FG525163); (**e**) *AcGA2ox1*: *GIBBERELLIC ACID OXIDASE 1* (Achn209941); (**f**) *AcNAC6* (Achn289291); (**g**) *AcMADS1* (Achn061601) and (**h**) *AcbZIP1* (Achn135561). *AdACTIN* (EF063572) was used as the housekeeping gene and the expression of fruit at harvest (D0) was calibrated as 1. Values are means of three independent biological replicates. Error bars represent SE. Different letters indicate significant differences at *p* < 0.05. Symbols are D = Day, W = Week, NT = non–treated and PROP = propylene treatment

To verify the RNA–seq results, we selected 24 DEGs that are associated with fruit ripening responses based on previous reports as well as their functional annotations for further analysis by RT–qPCR. First, we examined the expression of 8 genes selected from DEGs that were up−regulated by both propylene and low temperature (Fig. 5). In this study, the expression of cell wall modification−related genes, *AcPG*, *AcEXP1* and *AcXET2* (*XYLOGLUCAN ENDOTRANSGLUCOSYLASE 2*) significantly increased in propylene−treated fruit as well as during storage at 5 °C (Fig. 5a, b, c). A starch degradation−related gene, *Acβ–AMY1* (*β–AMYLASE 1*) registered high expression in propylene−treated fruit, and during storage, its expression increased both at 5 °C and 20 °C (Fig. 5d). The expression of *AcGA2ox1* (*GIBBERELLIN–2–OXIDASE 1*) also significantly increased by > 80–fold in propylene–treated fruit at 5 d and during storage at 5 °C (Fig. 5e). Finally, the expression of genes encoding TFs, *AcNAC6*, *AcMADS1* and *AcbZIP1* significantly increased in response to propylene and during storage at 5 °C (Fig. 5f, g, h). The expression of all the above genes showed insignificant changes during storage of kiwifruit at 20 °C. Frequent 1 − MCP treatments failed to inhibit the increased expression of these genes in fruit at 5 °C, suggesting that their regulation by low temperature was independent of ethylene.

**Fig. 6:**
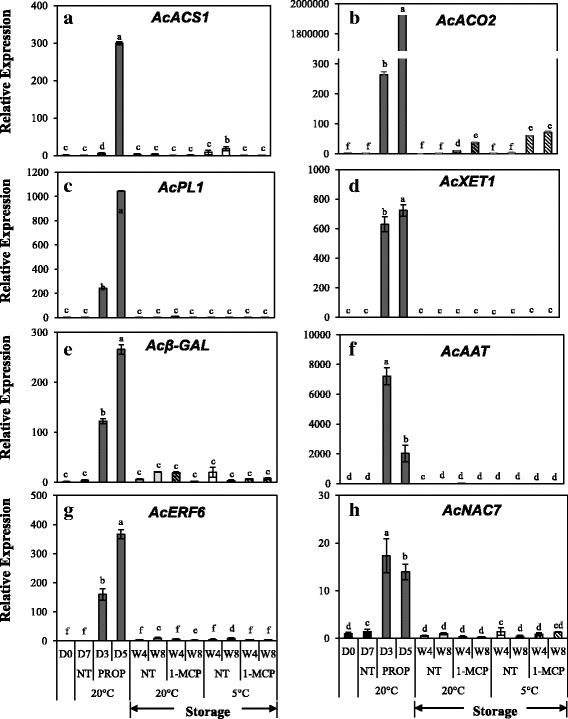
Reverse Transcriptase–Quantitative PCR analysis of selected kiwifruit genes that were exclusively up–regulated by propylene. Kiwifruit were continuously treated with 5000 μLL^− 1^ propylene (PROP) at 20 °C, alongside a non–treated group (NT). For storage, kiwifruit were kept at either 5 °C or 20 °C with (1–MCP) or without regular 1–MCP treatment (NT). 1–MCP was applied twice a week at 5 μLL^− 1^ for 12 h. Gene–specific primers were designed for (**a**) *AcACS1*: *ACC SYNTHASE 1* (Achn364251); (**b**) *AcACO2*: *ACC OXIDASE 2* (Achn326461); (**c**) *AcPL1*: *PECTATE LYASE 1* (Achn070291); (**d**) *AcXET1*: *XYLOGLUCAN ENDOTRANSGLUCOSYLASE 1* (Achn349851); (**e**) *Acβ–GAL*: *β*–*GALACTOSIDASE* (Achn123061); (**f**) *AcAAT*: *ALCOHOL ACYLTRANSFERASE* (Contig15634/ KJ626345); (**g**) *AcERF6* (GQ869857) and (**h**) *AcNAC7* (Achn104221). *AdACTIN* (EF063572) was used as the housekeeping gene and the expression of fruit at harvest (D0) was calibrated as 1. Values are means of three independent biological replicates. Error bars represent SE. Different letters indicate significant differences at p < 0.05. Symbols are D = Day, W = Week, NT = non–treated and PROP = propylene treatment

Next, we conducted RT–qPCR validation of 8 genes belonging to the category of DEGs that were exclusively regulated by propylene (Fig. 6). Out of the 13 genes annotated as *ACS* in the kiwifruit genome [16], only two were identified as DEGs and they were exclusively up−regulated by propylene (Additional file 6). *AcACS1* was selected for validation and its expression significantly increased by 300 − fold in propylene−treated fruit at 5 d while insignificant changes were recorded during storage (Fig. 6a). Furthermore, *AcACO2* expression drastically increased by ~ 260–fold and > 1,900,000–fold in propylene–treated fruit at 3 d and 5 d respectively, while its expression during storage was restricted to < 70–fold (Fig. 6b). Cell wall modification−related genes, *AcPL1*, *AcXET1* and *Acβ–GAL* (*β*–*GALACTOSIDASE*) also registered increased expression in propylene−treated fruit with little or no change in expression during storage (Fig. 6c, d, e). Propylene−treated fruit registered a drastic increase in expression of aroma volatile−related *AcAAT* (*ALCOHOL ACYLTRANSFERASE*) while fruit during storage showed insignificant changes (Fig. 6f). There was also a significant increase in expression of genes encoding TFs, *AcERF6* and *AcNAC7* in propylene−treated fruit while we observed insignificant changes during storage (Fig. 6g, h). These results suggest that the effect of ethylene on ripening in kiwifruit is distinct from that of low temperature.

**Fig. 7:**
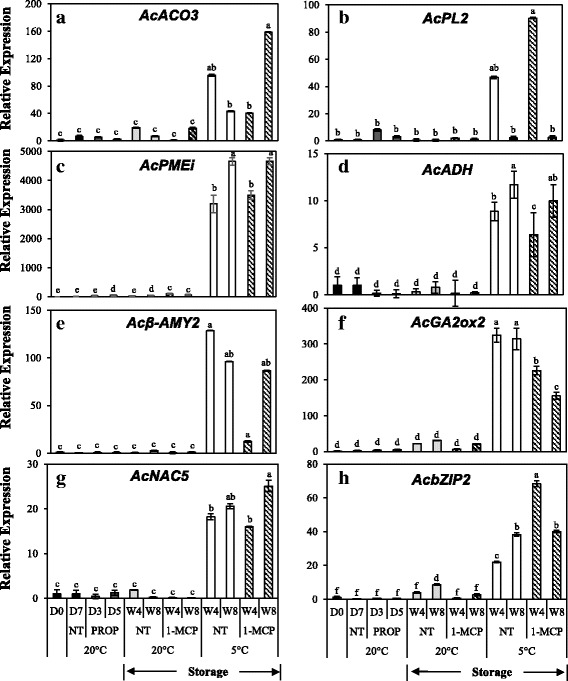
Reverse Transcriptase–Quantitative PCR analysis of selected kiwifruit genes that were exclusively up–regulated by low temperature. Kiwifruit were continuously treated with 5000 μLL^− 1^ propylene (PROP) at 20 °C, alongside a non–treated group (NT). For storage, kiwifruit were kept at either 5 °C or 20 °C with (1–MCP) or without regular 1–MCP treatment (NT). 1–MCP was applied twice a week at 5 μLL^− 1^ for ggmxf. Gene–specific primers were designed for (**a**) *AcACO3*: *ACC OXIDASE 3* (Achn150611); (**b**) *AcPL2*: *PECTATE LYASE 2* (Achn315151/HQ108112); (**c**) *AcPMEi*: *PECTIN METHYLESTERASE INHIBITOR* (Achn319051/FG458520); (**d**) *AcADH*: *ALCOHOL DEHYDROGENASE* (Achn262421); (**e**) *Acβ–AMY2*: *β–AMYLASE 2* (Achn212571); (**f**) *AcGA2ox2*: *GIBBERELLIC ACID OXIDASE 2* (Achn218871); (**g**) *AcNAC5* (Achn169421) and (**h**) *AcbZIP2* (Achn227711). *AdACTIN* (EF063572) was used as the housekeeping gene and the expression of fruit at harvest (D0) was calibrated as 1. Values are means of three independent biological replicates. Error bars represent SE. Different letters indicate significant differences at p < 0.05. Symbols are D = Day, W = Week, NT = non–treated and PROP = propylene treatment

To further confirm the distinct effect of low temperature on fruit ripening, we validated the expression of 8 genes obtained from the set of DEGs that were exclusively regulated by low temperature (Fig. 7). The expression of an ethylene biosynthesis−related gene, *AcACO3* showed insignificant changes in propylene−treated fruit while during storage, significantly higher expression was registered at 5 °C compared to 20 °C (Fig. 7a). We also observed higher expression of *AcPL2* and *AcPMEi* (*PECTIN METHYL ESTERASE INHIBITOR*) in fruit at 5 °C compared to 20 °C while insignificant changes were observed in propylene−treated fruit (Fig. 7b, c). The expression of *AcADH*, *Acβ–AMY2*, and *AcGA2ox2* was insignificant in propylene–treated fruit, while it markedly increased during storage at 5 °C (Fig. 7d, e, f). Finally, the expression of *AcNAC5* and *AcbZIP2* also registered significantly higher expression during storage at 5 °C compared to 20 °C while insignificant changes were observed in propylene−treated fruit (Fig. 7g, h). In this category of genes, frequent 1 − MCP treatments also failed to inhibit the increased expression at 5 °C, further supporting the hypothesis that they were regulated by low temperature independent of ethylene.

### Ripening behavior and expression of associated DEGs in on–vine kiwifruit

**Fig. 8:**
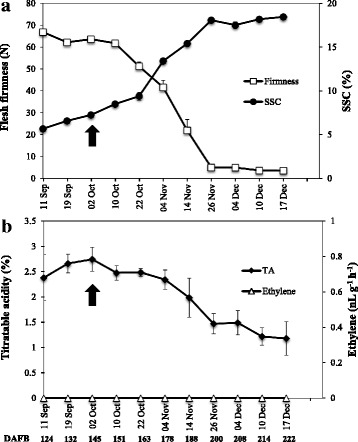
On–vine fruit ripening characteristics in kiwifruit. ‘Sanuki Gold’ kiwifruit were left attached to the vines after commercial harvesting date (indicated by the black arrow). Flesh firmness and SSC (**a**), ethylene production and titratable acidity (**b**) were determined using fruit that did not produce any detectable ethylene (five independent biological replicates). Error bars represent SE

Following the above findings, we monitored the ripening changes of ‘Sanuki Gold’ kiwifruit attached to the vines, and aligned them with the expression of the DEGs obtained from the RNA–seq analysis. For this purpose, kiwifruit were regularly harvested for a period ranging from 124 to 222 DAFB for determination of flesh firmness, SSC and TA. As indicated in Fig. 8A, flesh firmness of on–vine kiwifruit gradually decreased from ~ 66 N at 124 DAFB to ~ 61 N at 151 DAFB. This was followed by a sharp decrease to ~ 5 N at 200 DAFB and ~ 3 N at 222 DAFB. SSC of on–vine fruit steadily increased from ~ 5% at 124 DAFB to ~ 9% at 163 DAFB and afterwards, it rose sharply to a maximum of ~ 18% at 200 DAFB (Fig. 8a). Fruit TA showed a steady decrease from ~ 2.7% at 145 DAFB to ~ 1% at 222 DAFB (Fig. 8b). On–vine kiwifruit did not produce any detectable ethylene during the experimental period (Fig. 8b).

**Fig. 9:**
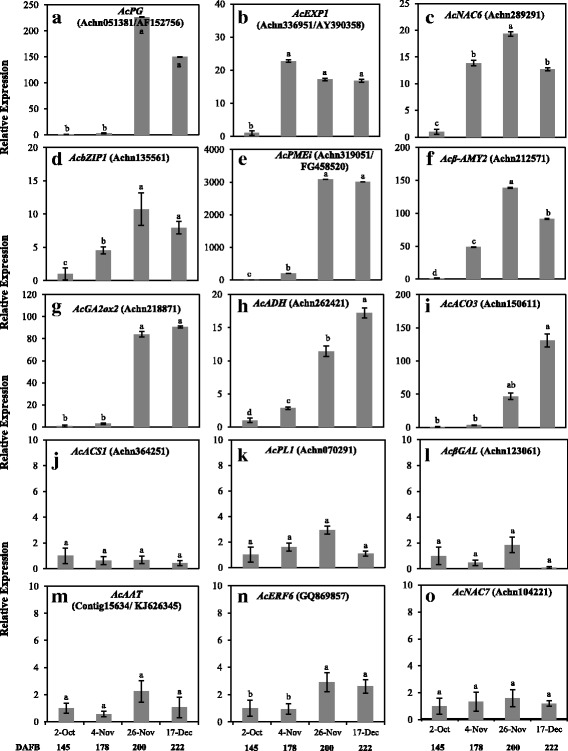
Reverse Transcriptase–Quantitative PCR analysis of selected genes during on–vine ripening in ‘Sanuki Gold’ kiwifruit. Genes for analysis were selected based on (i) DEGs that were up–regulated by both propylene and low temperature (**a, b, c, d**); (ii) DEGs that were exclusively up–regulated by low temperature (**e, f, g, h, i**) and (iii) DEGs that were exclusively up–regulated by propylene (**j, h, l, m, n, o**). *AdACTIN* (EF063572) was used as the housekeeping gene and the expression of fruit at harvest (D0) was calibrated as 1. Values are means of three independent biological replicates. Error bars represent SE. Values are means of three independent biological replicates. Error bars represent SE. Different letters indicate significant differences at p < 0.05

For analysis of gene expression, we extracted RNA from fruit at 145 DAFB when no ripening–associated changes (except for a small increase in SSC) were observed, and from fruit at 178, 200, and 222 DAFB during which fruit extensively ripened. First, we examined the expression of DEGs that were up−regulated by both propylene and low temperature. Among these, the expression of *AcPG* significantly increased from 200 DAFB while that of *AcEXP1*, *AcNAC6* and *AcbZIP1* increased from 178 DAFB (Fig. 9a, b, c, d). Next, we analyzed the expression of DEGs that were exclusively up−regulated by low temperature. In this case, *AcPMEi*, *Acβ–AMY2*, and *AcADH* expression significantly increased from 178 DAFB while *AcGA2ox2* and *AcACO3* expression increased from 200 DAFB (Fig. 9e, f, g, h, i). By contrast, the expression of DEGs that were exclusively up−regulated by propylene, *AcACS1*, *AcPL1*, *Acβ–GAL*, *AcAAT*, *AcERF6* and *AcNAC7* showed insignificant changes in expression during on−vine ripening in kiwifruit (Fig. 9j, k, l, m, n, o). Together, these results indicate that on−vine kiwifruit is similar to low temperature − modulated ripening during storage, while it was dissimilar to ethylene–dependent ripening.

## Discussion

### Healthy kiwifruit at 20 °C do not produce detectable ethylene at least for 56 days

Fruit ripening in climacteric fruit is marked by a coordinated ethylene burst (system II ethylene), which is believed to regulate the ripening process [1–3]. Several studies have reported a synchronized ethylene burst in a batch of kiwifruit within a few days after harvest [10, 33–35]. This study, however, demonstrated that the synchronized ethylene bursts observed in grouped kiwifruit is due to disease infections (Fig. 1). During grouped storage, a few fruit that exhibited disease symptoms initially produced ethylene, which in turn induced system II ethylene production in adjoining fruit (Fig. 1b). This was further confirmed by the finding that fungicide treatment delayed the onset of disease symptoms, and synchronized ethylene bursts (Fig. 1c). Fungicide application in postharvest kiwifruit only reduces the amount of fungal inoculum [36], and this could account for occurrence of disease infections and subsequent ethylene induction even in fungicide treated fruit.

In addition, some studies have reported that ripening and ethylene production in a population of kiwifruit is asynchronous [37]. This has been attributed to great variations in maturity stage among the individual fruit. In this study, however, ‘Sanuki Gold’ kiwifruit at the same maturity stage (SSC of 6–7%) depicted great variations in ethylene induction when stored individually (Fig. 1e, f). The few fruit that initiated ethylene production also depicted disease symptoms, consistent with the observations in grouped storage. We further demonstrated that > 50% of kiwifruit did not initiate ethylene production during the entire storage period (Fig. 1f); these fruit did not show any symptoms of disease infection. Overall, these findings indicate that the variations observed in system II ethylene production are not due to differences in maturity stage, but they are caused by disease infection. Thus, to avoid the effects of exogenous ethylene emanating from disease–infected fruit, it is important to provide adequate spacing between individual fruit coupled with active removal of fruit producing detectable ethylene (individual storage technique). In the present study, we applied this technique in further experiments to unravel ethylene–independent ripening mechanisms in kiwifruit.

### Ethylene−dependent and low temperature − modulated ripening mechanisms are independent in kiwifruit

The acceleration of fruit ripening in kiwifruit by ethylene or propylene has been reported in previous studies [38–41]. Our results in the present study are consistent with these reports since propylene exposure induced kiwifruit softening, SSC increase, reduction of TA and endogenous ethylene production (Fig. 2b, c, d, Additional file 1). The fact that these changes occurred within 5 d illustrates the powerful effect of the ethylene signal in triggering ripening in kiwifruit.

Despite showing a clear climacteric ripening behavior in response to exogenous ethylene or propylene, many studies have reported that most of the ripening−associated changes in kiwifruit during storage occur in the absence of any detectable ethylene [26, 27, 42]. In this study, we also observed significant softening, reduction of TA and SSC increase in kiwifruit that did not produce any detectable ethylene during storage at both 5 °C and 20 °C (Fig. 3, Additional file 1). Unlike ethylene–dependent ripening, information regarding the mechanisms regulating non–ethylene ripening in kiwifruit is limited. In this study, we demonstrated that low temperature is involved in the modulation of non–ethylene kiwifruit ripening during cold storage.

The gradual softening of kiwifruit during cold storage in the absence of detectable ethylene has been attributed to ethylene signaling, since kiwifruit are known to be extremely sensitive to low concentrations of ethylene [28, 29], and/or basal levels of system I ethylene present in most fruit [27]. In this study, we investigated the involvement of an ethylene signal through comparisons between the ripening behavior of kiwifruit during low temperature and room temperature storage. Interestingly, in kiwifruit that did not produce any detectable endogenous ethylene, softening and TA reduction occurred faster during storage at 5 °C compared to 20 °C (Fig. 3, Additional file 1). If these changes were triggered by system I ethylene, we would expect faster ripening in fruit at 20 °C compared to 5 °C as stipulated by temperature kinetics [43]. However, our results were inconsistent with this principle, indicating that low temperature acceleration of fruit ripening in kiwifruit was independent of the ethylene signal.

One approach that is used to study ethylene–dependent fruit ripening involves suppression of the ethylene signal by inhibitors, such as 1 − MCP. It was demonstrated that 1 − MCP inhibits ethylene action through irreversible binding and phosphorylation of ethylene receptors [21]. Previously, we reported that a single overnight exposure of ‘Sanuki Gold’ kiwifruit to 1–MCP made them insensitive to ethylene for at least 5 d [32]. In this study, frequent treatment of kiwifruit with 1 − MCP (twice a week) did not inhibit the accelerated ripening−associated changes during storage at 5 °C, providing further evidence for a distinct ethylene–independent role of low temperature in modulation of fruit ripening in kiwifruit.

Fruit ripening is genetically regulated by thousands of genes that control various processes such as ethylene biosynthesis, softening, pigment synthesis and degradation, accumulation of sugars, and release of volatiles [1, 3]. In kiwifruit, ethylene–induced modulation of genes associated with ethylene biosynthesis and signaling [10], fruit softening [32, 44], changes in SSC [45] and aroma volatile synthesis [46] has been previously demonstrated. This is consistent with our observation that the largest proportion of DEGs were induced by propylene (Fig. 4), accounting for the rapid ripening−associated changes recorded (Fig. 2). However, reports that discuss low temperature − modulation of ripening−associated genes in kiwifruit are limited. In this study, we demonstrated that numerous ripening−associated genes were triggered during storage of kiwifruit at 5 °C (Fig. 4), coinciding with the observed accelerated softening and reduction of TA (Fig. 3). We further demonstrated that ripening−associated genes in kiwifruit fall into three main groups. The first group comprises genes that are regulated by either ethylene or low temperature (Fig. 5, Additional file 4), suggesting that they are responsible for ripening−associated changes regardless of how they are induced. The second group is exclusively induced by ethylene (Fig. 6, Additional file 2), suggesting that they are specific to ethylene−dependent ripening in kiwifruit. The last group is exclusively induced by low temperature (Fig. 7, Additional file 3), further supporting the existence of a distinct ripening mechanism that is modulated by low temperature.

Previous studies on molecular regulation of fruit ripening have revealed several TFs that act either downstream or upstream of the ethylene–signaling pathway [1, 2]. In tomatoes, fruit ripening has been reported to coincide with increased expression of *bZIP1* [47–49]. In kiwifruit, fruit ripening is linked to several *ERFs* [11], *AcSEP4/RIN* [16, 25], and *NACs* [50]. In this study, several TFs such as *AcNAC6*, *AcMADS1* and *AcbZIP1* were induced by either propylene or low temperature (Fig. 5f, g, h), suggesting their possible roles in both ethylene−induced and low temperature − modulated ripening mechanisms in kiwifruit. Conversely, the expression of TFs such as *AcERF6* and *AcNAC7* exclusively increased in propylene−treated fruit (Fig. 6g, h), suggesting a mechanism by which the specific ethylene−dependent ripening pathway is regulated. Finally, several TFs such as *AcNAC5* and *AcbZIP2* were exclusively induced at 5 °C (Fig. 7g, h), suggesting their possible specific role in low temperature–modulated fruit ripening in kiwifruit. However, further research is required to ascertain the interactions of the TFs identified in this study with the ripening–associated genes in kiwifruit.

In winter pears such as ‘Passe Crassane’, a chilling treatment is required to induce ethylene production and subsequent fruit ripening [51, 52], demonstrating synergistic interactions between low temperature and ethylene signaling. However, in kiwifruit, the reduction of storage temperature retarded the rate of ripening and prolonged the number of days to ripening in propylene–treated fruit [53, 54]. Although the present study demonstrated that exposure of kiwifruit to either ethylene/propylene or low temperature resulted in eating–ripe fruit (Figs. 2 and 3, Additional file 1), it remains unclear whether these stimuli are complementary or self–sufficient. Therefore, it is reasonable to speculate that both ethylene and low temperature stimuli might be necessary for kiwifruit to achieve optimal ripening characteristics especially the aroma volatile profile, one of the cardinal quality criteria for ripe fruit. Further research is required to address this question.

It is also plausible to speculate that low temperature–modulated fruit ripening in kiwifruit is related with response to cold stress. In chilling–sensitive plants, cold stress resulting from extended low temperature treatments induce chilling injury symptoms such as tissue browning, woolly or dry texture, and abnormal cell metabolism such as membrane permeability disorders culminating in ethylene production [55]. To avoid development of chilling injury, the recommended commercial storage temperature for kiwifruit is usually 0–4 °C [42]. In this study, kiwifruit were stored at 5 °C, and during storage, fruit did not show any symptoms of chilling injury. In a separate study, we demonstrated faster softening and induction of associated genes in ‘Rainbow Red’ kiwifruit during storage at 15 °C, compared to 22 °C (Mitalo et al., unpublished). Storage at 15 °C can neither induce cold stress nor chilling injury symptoms in kiwifruit. These findings clearly indicate that the accelerated ripening of kiwifruit during low temperature storage is not due to cold stress. However, a clear distinction between low temperature–modulated ripening and cold stress related events might be hard to be accomplished, and ripening events that are independent of both ethylene and low temperature could exist in kiwifruit. Further research is required to expound this phenomenon.

### On–vine ripening in kiwifruit is a response to low temperature independent of ethylene

In this study, kiwifruit attached to the vines exhibited extensive ripening although endogenous ethylene production was not detected (Fig. 8), which is consistent with previous reports [16, 25]. This ripening pattern is commonly referred to as Phase 1 ripening [25], and has also been attributed to basal levels of system I ethylene and/or changes in ethylene sensitivity [16]. In this study, the gene expression patterns of fruit attached to the vines and those treated with propylene were quite dissimilar (Fig. 9j, k, l, m, n, o), suggesting that ethylene is not involved in regulating the on–vine ripening process. However, we observed a strong similarity in gene expression patterns between kiwifruit stored at 5 °C and those attached to the vines, suggesting that on–vine ripening could be a response to low temperature.

Separately, we demonstrated that a temperature < 10 °C is adequate for the induction of ripening in kiwifruit (Mitalo et al., unpublished). Maturation of kiwifruit occurs in autumn; the meteorological data indicated that environmental temperatures in the study area gradually decreased to < 10 °C in mid–October and ~ 5 °C in mid–November (Additional file 7). This suggests that on–vine kiwifruit responded to the decrease in environmental temperature, inducing ripening and associated genes. Previously, it was demonstrated that during Phase 1 ripening, kiwifruit lack the ability to produce endogenous ethylene due to repression of the system II ethylene–associated *SEP4/RIN* [16]. Thus, our findings suggest that low temperature could provide an alternative signal for the induction of phase 1 ripening in on–vine kiwifruit independent of ethylene.

## Conclusions

**Fig. 10:**
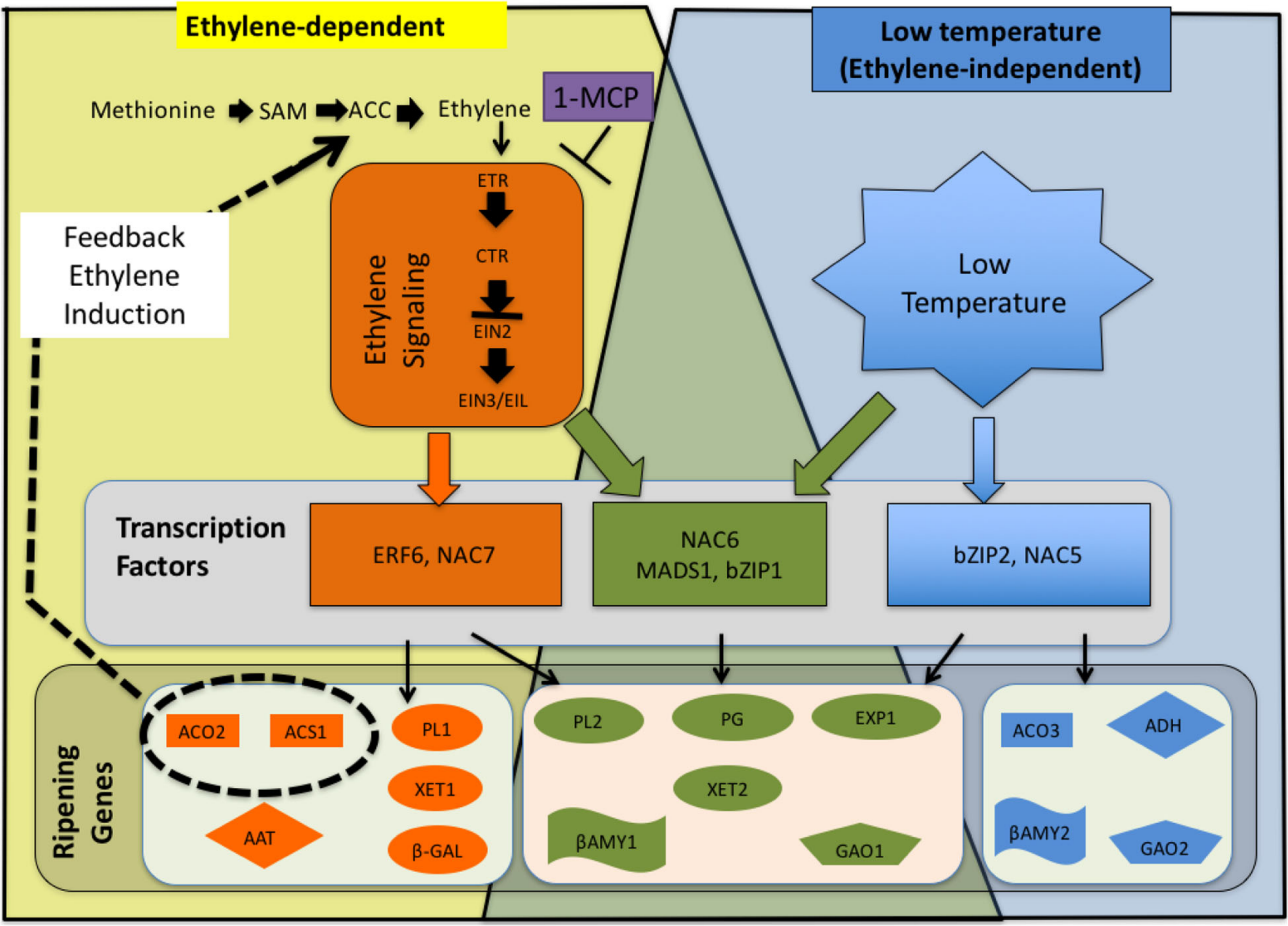
A simplified model showing ethylene–dependent and low temperature–modulated ripening pathways in kiwifruit. In ethylene–dependent ripening pathway, ethylene exclusively regulates the expression of specific genes (orange) as well as common genes (green). The expression of these ethylene–induced genes can be suppressed by 1–MCP application. Equally, low temperature regulates the expression of specific genes (blue) as well as common genes (green). Induction of these genes by low temperature is not suppressed by 1–MCP application, implying that they are independent of ethylene. Ripening–related gene classes are represented as rectangle (ethylene biosynthesis–related genes), oval (cell wall modification–related genes), wavy (carbohydrate metabolism–related genes), diamond (flavor–related genes), and pentagon (Gibberellin–2–oxidase genes)

Based on the results of this study, we propose a model showing the existence of two ripening pathways in kiwifruit (Fig. 10). The ethylene–dependent ripening pathway involves several genes that are either specific (orange) or common (green). Similarly, the low temperature − modulated ripening pathway involves several specific genes (blue) as well as the common ones (green). Thus, the present work provides a foundation for elaborating the control of these two ripening pathways in kiwifruit.

## Methods

### Plant material

‘Sanuki Gold’ and ‘Rainbow Red’ kiwifruit were obtained from a commercial orchard in Takamatsu, Japan while ‘Hayward’ kiwifruit were obtained from the experimental orchard at Okayama University, Japan. Fruit of each cultivar were harvested at commercial maturity stage corresponding to 131, 145 and 173 DAFB for ‘Rainbow Red’, ‘Sanuki Gold’ and ‘Hayward’ cultivars, respectively. All kiwifruit at this stage had an average SSC of about 6–7%. After harvest, all fruit were carefully screened to remove those producing ethylene, injured or with physical defects.

### Assessment of ethylene production during storage

Kiwifruit at commercial maturity (145 DAFB) were stored using either grouped or individual storage techniques. For grouped storage, fruit were placed into four containers (10 fruit per container) and the containers were covered lightly with a perforated polythene bag to reduce moisture loss. Separately, fruit were treated with fungicides before placing them into four containers, and every two weeks during storage. Fungicide treatment was conducted by dipping fruit into a mixed fungicide solution of 0.015 g/L oxytetracycline (Pfizer Co., Ltd., Japan), 0.15 g/L streptomycin (Pfizer), 0.5 g/L iprodione (FMC Chemicals Ltd., Japan), *Bacillus subtillis* HAI0404 spores (1 × 10^10^ cfu/L, Nippon Soda Co., Ltd., Japan), and 0.5 g/L benomyl (Sumitomo Chemical Ltd., Japan). In both setups, fruit were stored at 20 °C and ethylene production of individual fruit in each group was monitored twice a week.

For individual storage, 100 fruit were individually wrapped in perforated polythene bags and placed in containers while ensuring that they were ~ 10 cm apart from each other. The fruit were then stored at 20 °C and ethylene production of each fruit was monitored twice a week. A similar experiment was conducted using 80 fruit that were treated with 1–MCP twice a week. 1–MCP was released by adding water to the 1 − MCP powder (SmartFreshTM, Rohm and Hass, Philadelphia, PA, USA). Fruit were then exposed to 5 μLL^− 1^ of 1 − MCP for 12 h twice a week as described in our previous report [32].

### Determination of the effect of propylene

Kiwifruit were harvested at commercial maturity stage (145 DAFB) on October 2, 2012 and divided into two groups. The first group was continuously treated with 5000 μLL^− 1^ propylene at 20 °C to induce ethylene production, and to allow for determination of endogenous ethylene production [5, 41, 56]. The second group was held in air at 20 °C as a non–treated control. Sampling was done using five independent biological replications (five fruit) at 0 (harvest), 1, 3, 5, and 7 d for assessment of ethylene production, flesh firmness, SSC, and TA.

### Determination of the effect of storage temperature

Kiwifruit were harvested at commercial maturity stage (145 DAFB) on October 2, 2012 and divided into four groups. The first group was stored at 5 °C in air (non–treated) while the second group was treated with 1–MCP twice a week during storage at 5 °C in air (5 °C + 1–MCP). Similarly, the third group of fruit was stored at 20 °C in air (non–treated) while the fourth group was treated with 1–MCP twice a week during storage at 20 °C in air (20 °C + 1–MCP). 1–MCP treatment was done as previously described [32], to keep the fruit insensitive to ethylene throughout the storage period. Fruit in all treatments were individually separated by ~ 10 cm to allow for monitoring of ethylene production of each fruit. Any fruit that produced detectable ethylene (> 0.01 nLg^− 1^ h^− 1^) was transferred to separate storage chambers to prevent the ethylene from affecting adjoining fruit. These fruit exhibited a climacteric rise in ethylene production, developing rot disease symptoms within a few days of transfer. Therefore, only fruit that did not produce detectable ethylene (healthy fruit) were used for the analysis of fruit firmness, SSC, and TA at two–week intervals using 5 independent biological replications (five fruit).

### Evaluation of on–vine kiwifruit ripening

To monitor the on–vine ripening behavior, kiwifruit were harvested on 11 different occasions ranging from Sep. 11, Sep. 19, Oct. 2, Oct. 10, Oct. 22, Nov. 4, Nov. 14, Nov. 26, Dec. 4, Dec. 10 and Dec. 17 (corresponding to 124, 132, 145, 151,163, 178, 188, 200, 208, 214 and 222 DAFB respectively). At each harvest point, fruit were held at 20 °C for 24 h before determination of ethylene production. Only fruit that did not produce detectable ethylene were sampled for determination of flesh firmness, SSC, and TA using 5 independent biological replications (five fruit).

### Assessment of fruit ripening characteristics

Ethylene production was determined by incubating individual fruit in a 440–ml container for 2 h, after which 1 ml of headspace gas was withdrawn and injected into a gas chromatograph (Model–GC4 CMPF, Shimadzu, Kyoto, Japan), equipped with a flame ionization detector and an activated alumina column [32]. This procedure has a minimum ethylene detection capacity of 0.01 nLg^− 1^ h^− 1^. Flesh firmness was measured at two equatorial regions of the peeled flesh using a penetrometer (model SMT–T–50, Toyo Baldwin, Tokyo, Japan) fitted with an 8–mm plunger. SSC of the fruit juice was measured using a digital Atago PR–1 refractometer (Atago Co. Ltd, Tokyo, Japan) and the values were expressed as a percentage. TA was determined by titrating the fruit juice against 0.1 N NaOH and it was expressed as percentage citric acid equivalents (% TA).

### RNA extraction

Total RNA was extracted from ~ 3 g of ‘Sanuki Gold’ outer pericarp as previously described [57], using three independent biological replicates for each treatment. The extracted RNA was treated with DNaseI followed by RNA clean up using the FavorPrep after Tri–Reagent RNA Clean–up Kit (Favorgen Biotech co., Ping–Tung, Taiwan).

### RNA sequencing

cDNA libraries were constructed from RNA extracted from ‘Sanuki Gold’ kiwifruit samples at harvest (Day 0), treated with propylene for five days (Propylene), stored at 20 °C for four weeks with 1–MCP treatment (20 °C), and fruit stored at 5 °C for four weeks with 1–MCP treatment (5 °C). The libraries were sequenced using Illumina HiSeq 2500 (Hokkaido System Co., Ltd. Japan). Mapping and read count procedures were conducted using CLC genomic workbench (CLC Bio–Qiagen, Aarhus, Denmark) according to Akagi et al. [58]. After trimming, > 10 million reads were obtained for each sample and these were mapped to the genome of *A. chinensis* cv. ‘Hongyang’ as a reference [59]. Mapped reads of each gene were processed to reads per kb per million (RPKM) and false discovery rates (FDR) were calculated using a Bioconductor package for differential expression analysis edgeR [60]. DEGs were selected based on two criteria: (i) RPKM > 3.0 in at least one of the four sample groups (Harvest, Propylene, 5 °C, and 20 °C), (ii) > 3–fold increase or decrease in the average RPKM with FDR < 0.001 between Harvest and Propylene groups or 5 °C and 20 °C groups. RPKM values of each DEG were converted to fold changes and then used to generate a heat map using the CLC genomic workbench with Pearson’s correlation function displayed as the log annotation function (CLC Bio–Qiagen, Aarhus, Denmark).

### Reverse transcriptase–quantitative PCR analysis

Reverse Transcriptase–Quantitative PCR was conducted using cDNA of RNA extracted from samples at harvest (Day 0), non–treated fruit at Day 7, propylene–treated fruit at day 3 and 5, fruit stored at 5 °C or 20 °C for 4 and 8 weeks (both non–treated and 1–MCP treated). For monitoring gene expression on–vine, RNA was extracted from fruit harvested on Oct. 2, Nov. 4, Nov. 26 and Dec. 17 (145, 178, 200 and 222 DAFB respectively). First strand cDNA was synthesized from 2.4 μg RNA using RevTraAce reverse transcriptase (Toyobo, Osaka, Japan) and a random hexamer primer, according to the manufacturer’s instruction. Gene–specific primers (Additional file 8) were designed using Primer3 (version 0.4.0; http://bioinfo.ut.ee/primer3-0.4.0/). The reaction was performed using the MyiQ Single–Color Reverse Transcriptase–Quantitative PCR Detection System (Bio–Rad, Hercules, CA), according to manufacturer’s instructions. RT–qPCR conditions were set as; first step denaturing at 95 °C for 5 min, second step denaturing at 95 °C for 5 s, annealing and extension at 59 °C for 10 s (45 cycles). The specificity of all primers was verified by melting curve analysis. Relative expression values were then calculated as an average of 3 independent biological replications using *AdActin* as the housekeeping gene and mature fruit at harvest calibrated as 1.

### Statistical analysis

Data presented in this study was subjected to ANOVA followed by a post–hoc analysis using Duncan’s Multiple Range tests. The Heat map was constructed using a CLC genomics workbench with fold changes expressed as a log transformation with Pearson’s correlation analysis.

## Additional files

Additional file 1: Changes in fruit ripening characteristics of ‘Rainbow Red’ and ‘Hayward’ during storage at 20 °C and 5 °C with or without a 1–MCP treatment. Kiwifruit were harvested at commercial maturity and stored in containers, individually separated by about 10 cm. 1–MCP was applied twice a week at 5 μL L^− 1^ for 12 h. Flesh firmness (A), titratable acidity (B) and soluble solids content (C) were determined periodically using five independent biological replicates. Error bars represent SE. Different letters indicate significant differences at *p* < 0.05. (PPTX 63 kb)

Additional file 2: Genes that were exclusively regulated by propylene. Values for Harvest, Propylene, 20°CW4 and 5°CW4 indicate average RPKM of 3 independent biological replicates. Orange and blue color shading indicate genes that were up–regulated and down–regulated, respectively. (XLSX 378 kb)

Additional file 3: Genes that were exclusively regulated by low temperature. Values for Harvest, Propylene, 20°CW4 and 5°CW4 indicate average RPKM 3 independent biological replicates. Orange and blue color shading indicate genes that were up–regulated and down–regulated, respectively. (XLSX 198 kb)

Additional file 4: Genes that were regulated by either propylene or low temperature. Values for Harvest, Propylene, 20°CW4 and 5°CW4 indicate average RPKM of 3 independent biological replicates. Orange and blue color shading indicate genes that were up–regulated and down–regulated, respectively. (XLSX 179 kb)

Additional file 5: Genes that showed antagonistic expression patterns (either up–regulated by propylene but down–regulated by low temperature, or up–regulated by low temperature but down–regulated by propylene). Values for Harvest, Propylene, 20°CW4 and 5°CW4 indicate average RPKM of 3 independent biological replicates. Orange and blue color shading indicate genes that were up–regulated and down–regulated, respectively. (XLSX 157 kb)

Additional file 6: Selected ripening–associated genes that were differentially regulated either by ethylene or low temperature in ‘Sanuki Gold’ kiwifruit. (XLSX 33 kb)

Additional file 7: Average field temperatures in the experimental area during the 2012 kiwifruit growing season. Data were accessed from http://www.data.jma.go.jp/obd/stats/etrn/view/daily_s1.php?prec_no=72&block_no=47891&year=2014&month=12&day=&view=p1. (XLSX 37 kb)

Additional file 8 Kiwifruit gene–specific primers used for Reverse Transcriptase–Quantitative PCR. Accession numbers show genes sourced from the Kiwifruit Genome and NCBI Databases. Homolog genes are indicated in brackets. (XLSX 30 kb)

## Abbreviations

DAFB: Days after full bloom
RT–qPCR: Reverse Transcriptase–Quantitative PCR
