# Supplementary material for: Comparative transcriptome analysis reveals distinct ethylene–independent regulation of ripening in response to low temperature in kiwifruit
Source: BMC Plant Biol. 2018 Mar 21;18:47. doi: 10.1186/s12870-018-1264-y (PMC5863462; doi:10.1186/s12870-018-1264-y)
Supplement: Supplementary file 1 — Changes in fruit ripening characteristics of ‘Rainbow Red’ and ‘Hayward’ during storage at 20 °C and 5 °C with or without a 1–MCP treatment. Kiwifruit were harvested at commercial maturity and stored in containers, individually separated by about 10 cm. 1–MCP was applied twice a week at 5 μL L− 1 for 12 h. Flesh firmness (A), titratable acidity (B) and soluble solids content (C) were determined periodically using five independent biological replicates. Error bars represent SE. Different letters indicate significant differences at p < 0.05. (PPTX 63 kb) [file 12870_2018_1264_MOESM1_ESM.pptx]

## Slide 1
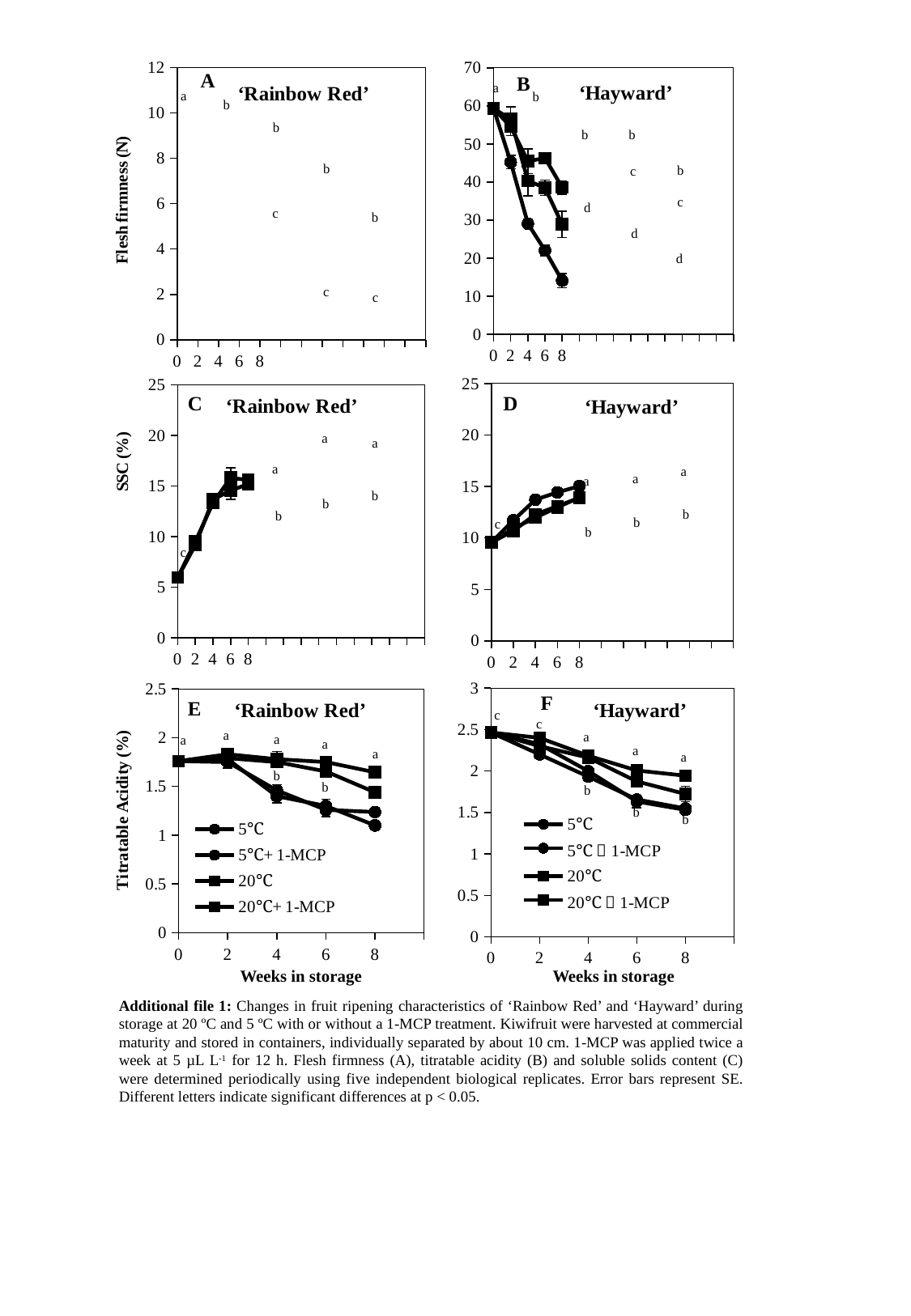

### Chart: ‘Hayward’
| Category | 5°C | 5°CM | 20°C | 20°CM |
|---|---|---|---|---|
| 0.0 | 59.3 | 59.3 | 59.3 | 59.3 |
| 2.0 | 46.21 | 45.21 | 56.51 | 54.51 |
| 4.0 | 28.87 | 29.04 | 40.33 | 45.49 |
| 6.0 | 18.35 | 22.0 | 38.46 | 46.27 |
| 8.0 | 14.93 | 14.08 | 28.85 | 38.56 |
| | None | None | None | None |
### Chart: ‘Rainbow Red’
| Category | 5°C | 5°C+1-MCP | 20°C | 20°C+1-MCP |
|---|---|---|---|---|
| 0.0 | 74.82 | 74.82 | 74.82 | 74.82 |
| 2.0 | 65.0 | 66.53 | 72.96000000000002 | 69.53 |
| 4.0 | 28.81000000000003 | 18.07999999999999 | 60.37 | 60.49 |
| 6.0 | 7.81 | 9.030000000000001 | 48.64 | 46.35 |
| 8.0 | 8.0 | 8.15 | 32.55 | 32.62000000000001 |
| | None | None | None | None |A
B
### Chart: ‘Rainbow Red’
| Category | 5℃ | 5℃+ 1-MCP | 20℃ | 20℃+ 1-MCP |
|---|---|---|---|---|
| 0.0 | 5.96 | 5.96 | 5.96 | 5.96 |
| 2.0 | 10.94 | 11.43333 | 9.526667000000002 | 9.173333000000001 |
| 4.0 | 14.7 | 15.17333 | 13.326667 | 13.66 |
| 6.0 | 16.85833299999987 | 16.38 | 15.81333 | 14.56 |
| 8.0 | 17.0 | 16.2 | 15.58333 | 15.18667 |
| | None | None | None | None |
### Chart: ‘Hayward’
| Category | 5℃ | 5℃+ 1-MCP | 20℃ | 20℃+ 1-MCP |
|---|---|---|---|---|
| 0.0 | 9.573333000000003 | 9.573333000000003 | 9.573333000000003 | 9.573333000000003 |
| 2.0 | 11.69333 | 11.71333 | 10.69333 | 10.92667 |
| 4.0 | 13.5 | 13.72 | 12.29167 | 11.98 |
| 6.0 | 14.44 | 14.42667 | 13.08 | 12.97333 |
| 8.0 | 14.8 | 15.04 | 13.9 | 13.93333 |
| | None | None | None | None |C
D
### Chart: ‘Rainbow Red’
| Category | 5℃ | 5℃+ 1-MCP | 20℃ | 20℃+ 1-MCP |
|---|---|---|---|---|
| 0.0 | 1.76 | 1.76 | 1.76 | 1.76 |
| 2.0 | 1.78 | 1.75 | 1.79 | 1.83 |
| 4.0 | 1.4 | 1.46 | 1.75 | 1.78 |
| 6.0 | 1.3 | 1.26 | 1.653999999999992 | 1.75 |
| 8.0 | 1.1 | 1.237899999999991 | 1.44 | 1.645 |
| | None | None | None | None |
### Chart: ‘Hayward’
| Category | 5℃ | 5℃＋1-MCP | 20℃ | 20℃＋1-MCP |
|---|---|---|---|---|
| 0.0 | 2.464 | 2.464 | 2.464 | 2.464 |
| 2.0 | 2.323 | 2.2 | 2.3 | 2.4 |
| 4.0 | 2.0 | 1.932000000000002 | 2.161249999999998 | 2.184 |
| 6.0 | 1.63285761095222 | 1.65678889677237 | 1.8747440172101 | 2.006692211909944 |
| 8.0 | 1.531345298824906 | 1.548691091867996 | 1.722809379312477 | 1.943312956501342 |
| | None | None | None | None |F
E
Weeks in storage
Weeks in storage
Additional file 1: Changes in fruit ripening characteristics of ‘Rainbow Red’ and ‘Hayward’ during storage at 20 ºC and 5 ºC with or without a 1-MCP treatment. Kiwifruit were harvested at commercial maturity and stored in containers, individually separated by about 10 cm. 1-MCP was applied twice a week at 5 µL L-1 for 12 h. Flesh firmness (A), titratable acidity (B) and soluble solids content (C) were determined periodically using five independent biological replicates. Error bars represent SE. Different letters indicate significant differences at p < 0.05.
a
a
b
b
b
b
b
b
b
c
c
d
c
b
d
d
c
c
a
a
a
a
a
a
b
b
b
b
b
c
b
c
c
c
a
a
a
a
a
a
a
a
b
b
b
b
b
